# Vestibular asymmetry predicts falls among elderly patients with multi- sensory dizziness

**DOI:** 10.1186/1471-2318-13-77

**Published:** 2013-07-22

**Authors:** Eva Ekvall Hansson, Måns Magnusson

**Affiliations:** 1 Department of Clinical Sciences in Malmö/Family Medicine, Lund University, Jan Waldenströms gata 35, SE 205 02, Malmö, Sweden; 2 Department of Clinical Sciences in Lund/ENT, Lund University, Skåne University Hospital, Lasarettsgatan 21, SE 222 85, Lund, Sweden

**Keywords:** Dizziness, Vestibular asymmetry, Falls, Prospective

## Abstract

**Background:**

Dizziness is the most common symptom in elderly patients and has been identified as a risk factor for falls. While BPPV is the most common cause of dizziness among elderly, multisensory deficits is the second, with visual, vestibular and proprioceptive reduced function. Asymmetric vestibular function is overrepresented in elderly persons with hip fractures and wrist fractures and can be accessed for screening.

The objective was to study if vestibular asymmetry, vibration sense, balances performance, postural sway in quiet stance and self-perceived handicap because of dizziness could predict falls among elderly, dizzy patients.

**Methods:**

In this prospective study with one year observation period, 55 patients (41 women, 14 men), 65 to 90 years old (median 80, interquartile range 11) with multisensory dizziness were included.

Vestibular function was screened with the headshake test and vibration sense was assessed using a tuning fork. Balance was assessed with four clinical measures and self-perceived dizziness handicap was assessed by the Dizziness Handicap Inventory. Postural sway was measured using a force plate.

**Results:**

Headshake test were pathologic in 24 patients, which substantially increased the risk of falls (OR 3.4). Thirteen of the 21 patients who had fallen (p = 0.03), and all 6 patients who sustained three falls or more (p = 0.04), had vestibular asymmetry. No other measure could predict the risk of falls (OR 0.55–1.71).

**Conclusion:**

Signs of vestibular asymmetry among elderly with multisensory dizziness could predict falls. Hence, it seems important to address fall-prevention programs to such a group of patients. Simple bedside tests of vestibular asymmetry might be a possibility to screen for one risk factor for falls among elderly.

## Background

Almost 45% of all outpatients with dizziness are seen in primary health care [1]. The prevalence of dizziness among elderly is substantial [2] and has been identified as a risk factor for falls [3]. The cause of dizziness is often benign, life-threatening conditions are rare [4] and the aetiology can often be assumed to be multifactorial [5]. The second most frequent cause of dizziness among elderly, after benign positional paroxysmal vertigo, is multisensory dizziness [6]. Multisensory dizziness can be defined as dizziness attributed to increasing age and deterioration of multiple sensory receptor systems [5,7].

Asymmetric vestibular function is overrepresented in elderly persons with hip fractures [8] and wrist fractures [9] and vestibular disorders may also be unrecognized as a cause to dizziness in the elderly [10].

Elderly people are highly dependent on somatosensory inputs to maintain postural control [11]. Sensory status in the lower limb has been recognized as important for postural control among healthy elderly persons [12] and older persons with polyneuropathy have been shown to have increased risk of falls [13]. Also, having poor ability to stand in tandem stance has been shown to double the risk of falls among elderly dizzy patients in primary health care [14] and postural sway, measured on a force plate, can give predictive values for falls [15]. Given this, elderly patients with multisensory dizziness may also have asymmetric vestibular function to a high degree, which might indicate a higher risk of falls.

The aim of this study was therefore to prospectively study if vestibular asymmetry, vibration sense, balance performance, postural sway in quiet stance and self-perceived handicap because of dizziness could predict falls among elderly, dizzy patients.

## Method

### Study population

Inclusion of patients was done from January 2007 to October 2011 and inclusion criteria were: age 65 years and above, with multisensory dizziness, i.e. dizziness attributed to age and deterioration of sensory receptor system [5,7]. Exclusion criteria were: younger than 65 years of age with other cause of dizziness than multisensory. These patients had all been referred to physiotherapy at a Primary Health Care Centre, in Malmö, Sweden. There are about and 20 health care centres in the area, covering a population of 270 000 inhabitants. The present centre acts as an internal referral centre for such patients and has a physiotherapist specialized in vertigo and dizziness and extended possibilities for diagnosis and treatments. All patients were also assessed with the Dix-Hallpike manoeuvre to exclude benign paroxysmal positional vertigo [16].

### Vestibular asymmetry

Vestibular asymmetry was assessed by passive head shaking, at a frequency of approximately 2 HZ/15 sec, in supine position, and performed with video-frenzels. Eye-movements were recorded on video and nystagmus was assessed by one of the authors (MM) who was blinded toward the status or diagnosis of the patients and recordings were mixed with recordings of normative data. The test was considered pathologic if nystagmus could be detected and the asymmetry be determined [12].

### Vibration sense

To measure vibration sense in the lower limb, a tuning fork (256 Hz) was used towards the base of the first metatarsal bone, the medial malleolus and the medial surface of the tibia levelled with the tibial tuberosity [12]. The test was considered as normal if the patient could feel vibrations at all three levels and as not normal if the patient not could feel vibrations in one, two or three of the levels.

### Clinical balance assessement

All patients were assessed with four different balance measures, which have been shown in previous studies to be relevant for assessing elderly dizzy patients in primary health care [17].

– Tandem stance with eyes open and with eyes closed [18]. Time up to 30 seconds was measured. Three attempts were allowed and the best attempt was used.

– Standing on one leg with eyes open (SOLEO) and standing one on leg with eyes closed (SOLEC) [18]. Time up to 30 seconds was measured and three attempts were allowed.

– Walking in the modified figure of eight [19]. The line in the figure is four centimetre broad and the inner circle has a diameter of 163 centimetres. The participants were instructed to walk twice in the figure, in their preferred walking speed and with preferred step-length. Steps outside the figure were counted.

– Walking heel to toe on a five-metre long and five-centimetre broad line [20], where steps outside the line were counted. The participants were asked to walk heel to toe in preferred speed.

– Walking as fast as possible for 30 m with one turn after 15 m [21]. Time in seconds was measured with a manual stop watch.

### Postural sway

Postural sway during quiet stance, with open and closed eyes for 30 s, was measured using a triangular force plate, with strain gauge transducers at each corner of the platform, a three-channel DC amplifier, en eight-channel analogue-to-digital converter and a computer program installed in a laptop PC (Good Balance™, Metitur Ltd, Finland, http://www.metitur.com). On the basis of the vertical force signals from each corner of the platform, the system calculated the mean speed of the movement of centre of pressure (CO) in mediolateral (ML) direction as well as in anteriorposterior (AP) direction (mm/s). Sway mean velocity moment (SMVM) (mm/sec) was also measured. Force plates have been tested for test-retest and intrasession reliability as well as validity [22,23].

### Self-perceived handicap

Self-perceived handicap because of dizziness was measured with the Dizziness Handicap Inventory (DHI) [24]. The inventory has been translated into Swedish and the Swedish version has been tested for reliability [25] and has proved to be appropriate for use in primary health care [17]. The inventory comprises 25 items, organized in three different dimensions: functional, emotional and physical. The score can be calculated as a whole but can also be divided into the three different dimensions. The total maximum score is 100 points; for the functional dimension it is 32 points, the emotional 40 points and the physical 28 points. The higher the score, the greater the level of self-perceived handicap.

### Falls

Falls were registered in follow-ups by means of interviews over telephone, to all patients after 3, 6, 9 and 12 months. On these occasions, the patients were asked whether or not they had fallen. A fall was defined as “an unexpected event in which the participants come to rest on the ground, floor, or lower level” [26]. The falls were registered in a classification system, Older Adult Service and Information System (OASIS) [27]. The system classifies falls into four different categories, extrinsic falls, intrinsic falls, non-bipedal falls and non-classifiable falls.

### Statistics

Logistic regression was used to calculate the odds ratio (OR) for falls. Since the measures had a skewed distribution, median values were used as cut-off value to dichotomize continuous data, creating one group with poor outcome and one with good. Proportions were calculated using chi^2^. Calculating with 50% more falls among patients with poor ability to stand in tandem stance [14] and with poor vibration sense in the lower limb [13], a power of 80% and the significance level set at 0.05, a sample size of 60 patients was required [28]. PAWS statistics 19.0 was used for the analysis (SPSS Inc, software location Lund University).

### Ethics

Participation in the study was strictly voluntary. All patients were informed about the study, both verbally and in writing, and gave their signed consent to participate. All patients were taken care of, both participants and non-participants. The measures used in the study are all clinical measures of no discomfort for the patients. The study was approved by the regional ethics review board in Lund.

## Results

**Figure 1:**
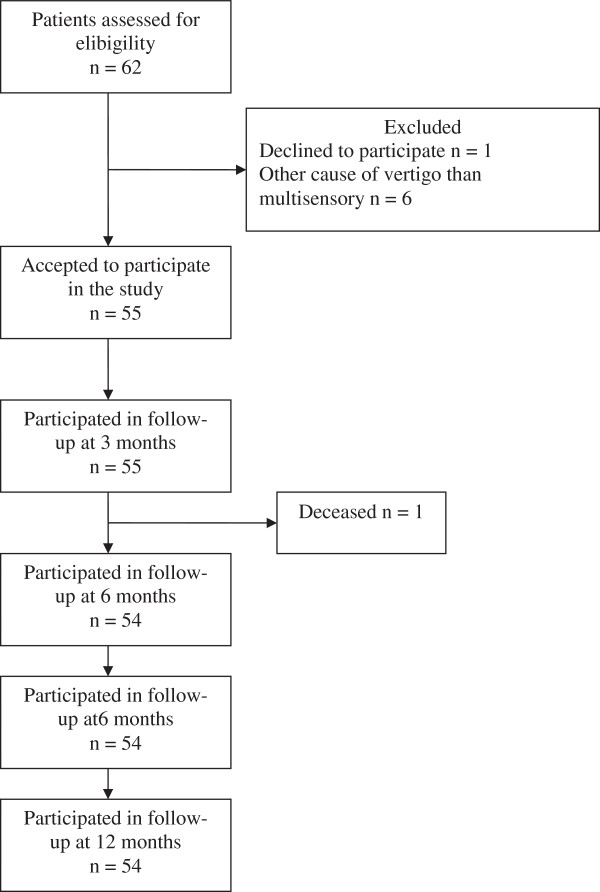
Flow chart of the study.

A total of 62 patients were assessed for eligibility in the study and 55 patients were included, 41 women and 14 men, 65 to 90 years old (median 80 years of age, interquartile range 11). A flow chart of the study is shown in Figure 1. Headshake test was pathologic in 24 patients, normal in 28 and undecided in 3 patients. Fifteen patients had disturbed vibration sense (Table 1). During the one year observation time, a total of 21 patients had fallen. Of those, 8 had a normal headshake test, 13 a pathologic (p = 0.03) and 6 had disturbed vibration sense (0.06). Of the fallers, two had both pathologic headshake-test and disturbed vibration sense. Eleven patients reported one fall, 4 patients reported 2 falls and 6 patients reported 3 falls or more. Among the latter, two had three falls, two had four falls, one had six falls and one patient reported seven falls. All patients who had fallen three times or more, had a pathologic headshake test (p = 0.04) on the previous inclusion assessment and both patients with the most falls, had additionally disturbed vibration sense. Five of the patients who had fallen sustained an injury that required health care and one of them fractured a vertebrae. Four of these five patients had a pathologic headshake test at inclusion.

**Table 1:** **Characteristics of the study population and odds ratio for falls**, **including confidence interval (CI)**

**Measures**	**Total group n = 55**	**OR (CI)**
Age. Median (IQR) *	80 (11)	1.37 (0.44–4.26)
Men/women	14/41	0.9 (0.28–3.55)
Headshake test (pos/neg/ undecided)	24/28/3	3.40 (1.09–10.59)**
Vibration sense (normal/not normal)	40/15	1.08 (0.56–2.05)

*Interquartile range ** Significant at the 0.05 level.

### Risk analysis

Having a pathologic headshake test increased the risk of falls substantially and significantly (OR 3.4) (Table 1). Poor outcome in dynamic balance measures (OR 1.25–1.57) seems to increase the risk of falls more than static balance measures (OR 0.77-–1.11) but none of these OR’s were statistically significant (Table 2). A high degree of postural sway in quiet stance, did not seem to increase the risk of falls (OR 0.55–1.22), neither when measured standing with open nor closed eyes (Table 2). A higher total score on DHI gave higher OR (2.28) for falls than the increased rating on the subscales (OR 1.08–1.71), but none of these correlated to a statistically significant increased risk of falls (Table 2).

**Table 2:** Median values and interquartile range (IQR) for balance measures, postural sway, self-perceived handicap because of dizziness and odds ratio for falls, including confidence interval (CI)

**Measure**	**Total group n = 55 (IQR)**	**OR (CI)**
Tandem stance eo* (sec)	19 (27)	1.00 (0.33–3.02)
Tandem stance ec (sec)	2 (4)	0.92 (0.30–2.79)
SOLEO (sec)	3.5 (7.5)	0.77 (0.31–1.92)
SOLEC (sec)	1.0 (2)	1.01 (0.35–3.43)
Figure of eight (steps outside the line)	12 (14.25)	1.25 (0.39–3.94)
Walking heel to toe (steps outside the line)	7 (8.5)	1.43 (0.45–4.54)
30 m walk (sec)	29 (12.25)	1.57 (0.49–5.01)
Mediolateral sway eyes open (sway velocity in mm/sec)	8.35 (7.75)	1.22 (0.35–4.19)
Anteriorposterior sway eyes open (sway velocity in mm/sec)	12.35 (10.15)	0.75 (0.35–4.18)
Sway mean velocity moment (mm/sec)	30.45 (39.85)	0.82 (0.24–2.82)
Mediolateral sway eyes closed (sway velocity in mm/sec)	16.55 (17.6)	0.61 (0.19–1.90)
Anteriorposterior sway eyes closed (sway velocity in mm/sec)	24.30 (21.55)	0.55 (0.16–1.91)
Sway mean velocity moment eyes closed (mm/sec)	90.75 (167.4)	1.18 (0.33–4.19)
DHI total score (median points)	42 (28.5)	2.28 (0.68–7.69)
DHI functional score (median points)	13 (14)	1.22 (0.39–3.79)
DHI emotional score (median points)	14 (8)	1.08 (0.35–3.38)
DHI physical score (median points)	13 (10.5)	1.71 (0.54–5.41)

* *eo* eyes open, *ec* eyes closed, *SOLEO* standing one leg eyes open, *SOLEC* standing one leg eyes closed.

## Discussion

This study suggests that assessing vestibular asymmetry could predict falls among elderly patients with multisensory dizziness. During the observation time, three falls or more only occurred among patients with vestibular asymmetry.

The video recorded headshake test in our study was assessed blindly by one of the authors (MM), who is experienced in otoneurology. We therefore believe that the findings of pathologic or normal headshake-test are sound. In our study, 24 out of 55 elderly persons with multisensory dizziness had vestibular asymmetry. This is, as expected, higher than in an ageing normal population where an asymmetry was found in 18 out of 49 elderly healthy persons [12]. However, it was lower than among elderly patients with fractures of the hip or wrist [8,9].

Elderly seem to rely more on vision than younger to maintain postural control [20]. Unfortunately, we had no possibility to assess vision acuity and therefore do not know if that influenced falls in this group of patients. It should however be considered that not only visual acuity but also visual motion perception is affected by age and may be of even more importance. Such motion perception and responses have also been shown to be affected by a vestibular asymmetry [29-31]. Also, the present primary health care centre acts as an internal referral centre for patients with vertigo and dizziness and therefore has extended possibilities for diagnosis and treatments of vertigo and dizziness. We therefore believe that the diagnosis of multisensory dizziness is valid. Sample size in the study is small, which has to be considered when interpreting the results.

We followed the patients for one year, by means of telephone calls every third month. Time periods of three months seems to be a reasonable time for recollection of falls in such a population and has been used in other studies [14]. Also, telephone calls every third month has showed to have good agreement when compared to falls calendars [32]. It should be pointed out that none of the included patients had any obvious communicative impairment.

Among elderly with diabetic neuropathy a prominent decline in balance has been reported [33]. We had therefore expected that patients, who had decline in vibration sense in the lower leg, would have a higher risk of falls, but this was not the case (OR 1.08). Only 15 patients were found to have a decline in vibration sense which might explain the lack of correlation.

Dizziness may also lead to impaired health-related quality of life [34]. We measured self-perceived handicap because of dizziness with DHI. Our study group had similar median values on DHI and on clinical balance measures to those of another study, including elderly patients with multisensory dizziness [17].

There seems to be difficulties in the ability to identify patients at high risk for falling in hospital settings [35]. However, elderly patients with dizziness who are admitted to hospital have been shown to be at high risk of suffering from any kind of fracture regardless of the reason for admission [36]. It therefore seems important to address fall-prevention programmes towards patient groups with increased risk of falls, such as elderly patients with multisensory dizziness and vestibular asymmetry. For example, simple bedside vestibular test like the headshake test might add valuable information on prediction of falls. In such cases actions to treat the vestibular asymmetry, by applying vestibular rehabilitation or balance training, might be beneficial. However, more research on larger populations is needed to ascertain such assumptions.

## Conclusion

Signs of vestibular asymmetry could predict falls among elderly persons with dizziness. Hence, it seems important to address fall-prevention programs to such a group of patients. Also, simple bedside tests of vestibular asymmetry might be a possibility to screen for at least one risk factor for falls among elderly.

## Competing interests

We did not have any external founding for this research and there are no competing interests.

## Authors’ contribution

EEH has contributed in designing the study, in data-collection and performed the statistical analyses of data and has contributed in writing the manuscript. MM has contributed in designing the study, in analyses of data and has contributed in writing the manuscript. Both authors has read and approved the final manuscript.

## Pre-publication history

The pre-publication history for this paper can be accessed here:

http://www.biomedcentral.com/1471-2318/13/77/prepub
